# Effect of Sodium Chloride on Aggregation of Merocyanine 540 and Photosensitized Inactivation of Staphylococcus aureus and Pseudomonas aeruginosa

**Published:** 2011

**Authors:** T.A. Shmigol, V.A. Bekhalo, Е.V. Sysolyatina, E.V. Nagurskaya, S.A. Ermolaeva, A.Ya. Potapenko

**Affiliations:** Pirogov Russian National Research Medical University; Gamaleya Research Institute of Epidemiology and Microbiology, Ministry of Health and Social Development of the Russian Federation

**Keywords:** antimicrobial photodynamic therapy, merocyanine 540, *Staphylococcus aureus*, *Pseudomonas aeruginosa*

## Abstract

Merocyanine 540 (MC540) is used as a photosensitizer for the inactivation of microorganisms. The following is already known about MC540: firstly, MC540 exists in distilled water in both monomeric and dimeric forms, and the addition of salts into a MC540 solution leads to the formation of large aggregates that can be detected by the resonance light scattering technique. Secondly, singlet oxygen can only be photogenerated by MC540 monomers. In the present work, we studied the effect of MC540 in the aggregated state on the rate of photosensitized inactivation of*Staphylococcus aureus*and*Pseudomonas aeruginosa*. To this end, bacteria either in MC540-containing distilled water or in a 0.25 M sodium chloride aqueous solution also containing MC540 are irradiated (546 nm). The results show that, in the presence of salt, the aggregation of MC540 greatly increases the efficiency of the MC540-photosensitized inactivation of*P. aeruginosa*and*S. aureus*. In the presence of salt, the rates of*P. aeruginosa*and*S. aureus*inactivation increase by factors of 10 and 30, respectively, in comparison with the rate of inactivation observed in the case of distilled water. Our results suggest that a salt-induced photosensitization mechanism can switch from the singlet oxygen to the free-radical pathway.

## INTRODUCTION

The search for effective methods of antibacterial protection has led to the development of antimicrobial photodynamic therapy. The photodynamic effect was first described by Raab in 1900, and the term “photodynamic reaction” was first introduced by Tappeiner in 1904 [1]. The photodynamic inactivation of bacteria occurs under the action of light in the presence of photo-sensitizers (FSs) and molecular oxygen. Through exposure to light, photosensitizers are activated, thereby producing free radicals or singlet oxygen, which are fatal to infectious agents.

MC540 is known to be capable of inactivating infectious agents [2–5]. This happens as a result of the occurrence of two types of photodynamic reactions: types I and II. In type I reactions, a photosensitizer in the triplet-excited state reacts directly with a substrate, but it does not react with molecular oxygen. During reactions of this type, the electron (or hydrogen) is transferred from a photosensitizer molecule found in the triplet-excited state to a substrate found in the ground state. Depending on the reacting pair, both transfer from a substrate to a photosensitizer and *vice versa* are possible. Free radicals are produced as a result of such reactions; oxygen enters into the reaction at later stages, thereby leading to the photooxidation of the substrate [6]. 

In type II reactions, the primary reaction occurs between a photosensitizer in the triplet-excited state and molecular oxygen. This type of reaction causes the occurrence of an excited singlet oxygen or superoxide radical anion. The main role in subsequent reactions is played by the singlet oxygen, which oxidizes a substrate. The detachment of an electron from the excited photosensitizer, accompanied by the formation of a superoxide anion, may also occur. In the subsequent reactions of substrate oxidation, only the superoxide anion and other active forms of oxygen participate [6].

In preliminary studies performed *in vitro* , MC540 aggregates were shown to photobleach faster than monomers and dimers [7, 8]. Resonance light scattering (RLS) is the most sensitive and selective method for studying aggregation processes in dyes. The principle of RLS lies in the drastic increase in Rayleigh light scattering in the region of the absorption band of aggregated dye molecules. The above-mentioned phenomenon is typical of strongly absorbing chromophores that form large aggregates in which exciton interaction between the π-electron systems of dye molecules occurs [9]. 

Herein, we describe the effect of sodium chloride on the aggregation of MC540, on the rate of its photobleaching, and on the photosensitized inactivation of *Staphylococcus aureus* and *Pseudomonas aeruginosa* bacteria. 

## EXPERIMENTAL

**Reagents**

**Fig. 1:**
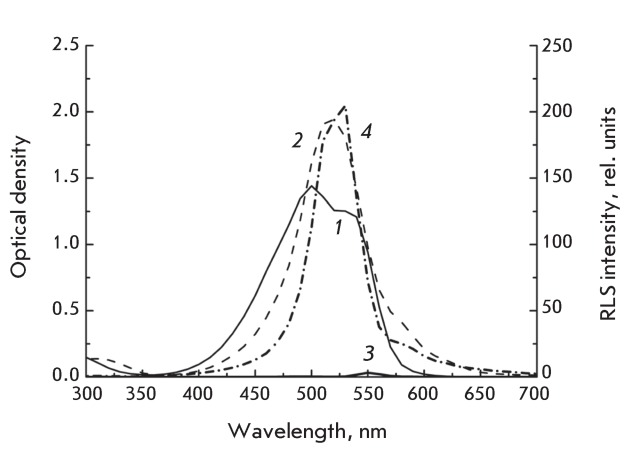
Absorption ( *1* ,  *2* ) and resonance light scattering ( *3* ,  *4* ) spectra of a 25 μM MC540 in distilled water ( *1* ,  *3* ) solution and in 0.25 M NaCl solution ( *2* , *4* ).

**Fig. 2:**
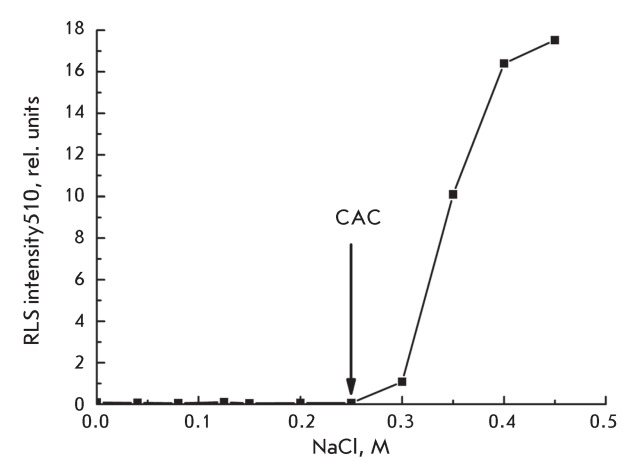
Dependence of the RLS intensity at 510 nm in a 7.6 μM MC540 solution on the NaCl concentration. An arrow indicates the critical aggregation concentration (CAC) of NaCl above which formation of MC540 aggregates occurs; the latter are detected by the RLS technique.

 Merocyanine 540 (Sigma, USA) and chemically pure NaCl (REAKHIM, Russia) were used. A stock solution of MC540 (10 ^–3^  M) in distilled water was prepared in the day of the experiment. Working solutions (25 µM) were obtained by diluting the stock solution with water or with a 0.25 M NaCl solution. 

Merocyanine 540 solutions and suspensions of microorganisms were irradiated with light generated by a DRSh-250 mercury quartz lamp (Zelenograd, Russia). Monochromatic light was obtained via a 546-nm glass filter (Russia).

The intensity of light was measured using an IM-1-2 photodiode (Russia) calibrated for 546 nm. The irradiation of MC540 solutions was performed in cells with a thickness of 1 cm under side lighting and continuous stirring with a magnetic stirrer at a temperature of 23°C.

The absorption spectra were measured using a Shimadzu UV-1601 PC spectrophotometer (Japan).

The resonance light scattering spectra were measured using a Shimadzu RF-1501 Spectrofluorimeter (Japan). The spectral measurements were performed using quartz cells with a thickness of 1 cm. The measured RLS spectra were corrected taking into account the effects of the inner optical filtering effect and the sensitivity of the instrument, in accordance with the procedure described in [10] by Tikhomirov *et al* . 

**Cell Cultures**

 In this work, we used *S. aureus * and *P. aeruginosa* clinical isolates of strains 78 ( *Sa78* ) and 104 ( *Pa104* ), respectively. The microorganisms were taken from the collection of the Gamaleya Research Institute of Epidemiology and Microbiology of the Ministry of Health and Social Development of the Russian Federation. 

**Preparation of Cell Suspension **

*S. aureus * and *P. aeruginosa* were incubated in a brain heart infusion broth (Difco, USA) for 12 h at 37°C and then diluted in a phosphate-buffered saline solution to an optical density value ( *D*_600_ ) of 1, which corresponds to a concentration of 10 ^9^ CFU/ml. The bacterial suspension (1 ml) was washed twice by centrifugation in sterile distilled water (7000 rot/min, 3 min) and then resuspended in 10 ml of sterile distilled water. 

In order to obtain samples for irradiation, a 50 µM solution of MC540 in distilled water was mixed with the bacterial suspension, which was prepared according to the aforementioned process, at a ratio of 1: 1. The 25 µM MC540 sample in a salt solution was prepared by mixing 100 µM MC540, 1 M NaCl, and the bacterial suspension at a ratio of 1 : 1 : 2, respectively. Prior to irradiation, a MC540 solution with a final concentration of 25 µM was incubated with cells for 10 min in the dark at room temperature. Following irradiation, the sample underwent a series of 10-fold dilutions in a GRM-1 agar medium (Obolensk, Russia) and was poured into Petri dishes. Both the treated and control samples were incubated in a thermostat at 37°C. The amount of grown colonies was calculated after 24 h. The bactericidal effect was determined as a ratio of the survived bacteria in the experimental to the control groups, respectively.

**Kinetics of Photobleaching of MC540 and Photoinactivation of Bacteria**

**Fig. 3:**
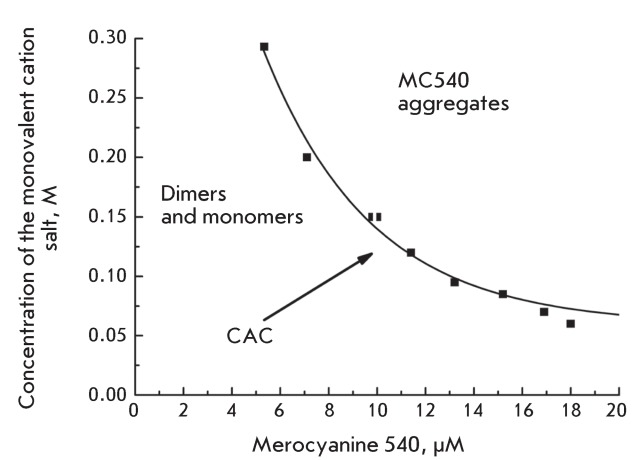
Dependence of the critical aggregation concentration (CAC) for monovalent cation salts on the MC540 concentration.

**Fig. 4:**
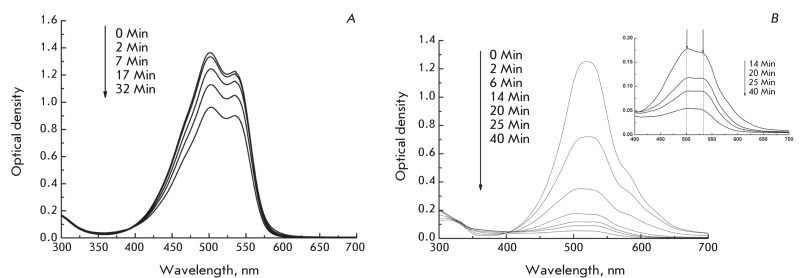
Photobleaching of MC540 (25 µM) in distilled water ( *A* ) and in NaCl containing (0.25 M) solutions ( *B* ).

 A kinetic analysis of the photobleaching of 25 μM MC540 in the distilled water solution and the sodium chloride (0.25 M) solution was carried out for the initial range of the dose-dependence curve (at which decay of the photosensitizer has a mono exponential behavior) plotted in semilogarithmic coordinates. The photobleaching constant (m ^2^ /kJ) was ascertained according to the following: *k*  = ln( *D* / *D*_0_ ), where *D*_0_ and *D* are the optical densities at a wavelength of 518 nm in the initial moment of time and under irradiation at a dose of *F* (kJ/m ^2^ ), respectively. 

A kinetic analysis of the photoinactivation of *P. aeruginosa * and *S. aureus * bacteria in a the 25 µM MC540 distilled water and sodium chloride (0.25 M) solutions was performed for the initial segment of the dose-dependence curve constructed in semi-logarithmic coordinates. The photoinactivation constant was determined in accordance with the following formula: 

*β* = ln( *CFU* / *CFU*_0_ )/ *F,*

where *CFU*_0 _ and *CFU* are the parameters that characterize the ability to form colonies at the initial time and under irradiation at a dose of *F* (kJ/m ^2^ ), respectively. 

**Statistical Analysis**

 The mean values for the magnitudes studied were determined, and their standard errors of mean were estimated as follows: 
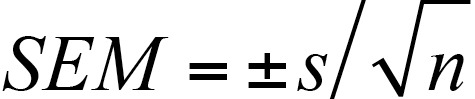
, where *s * is the sample standard deviation. 

## RESULTS

**Absorption Spectra of MC540**

**Fig. 5:**
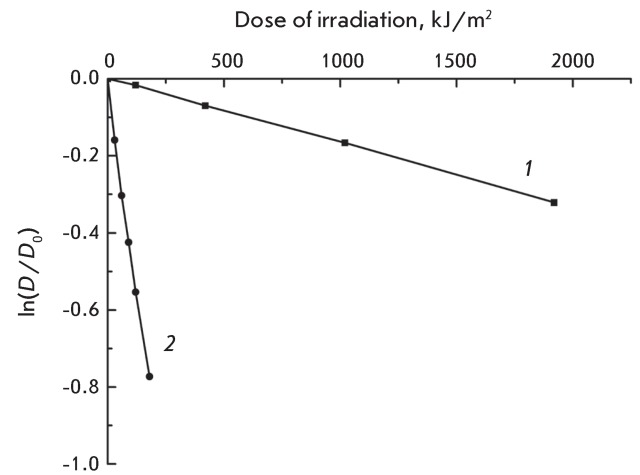
Dose-response curves of photobleaching of a 25 μM MC540 in distilled water ( *1* ) and in NaCl-containing (0.25 M) solutions ( *2* ). *D* and *D *_0_ are the optical densities at 518 nm measured in irradiated and nonirradiated solutions, respectively.

**Fig. 6:**
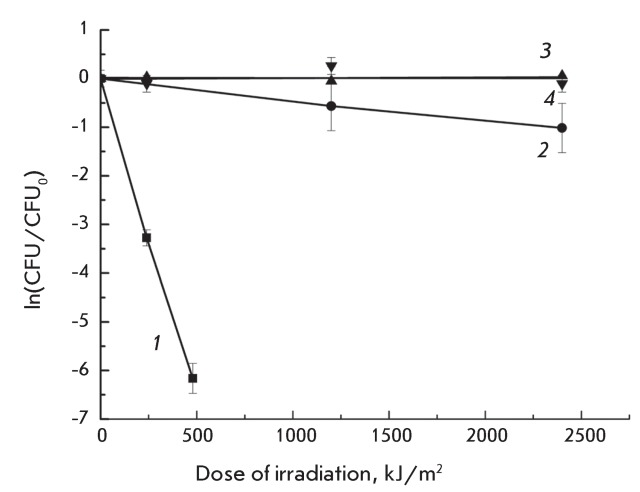
Dose-response curves of *S. aureus* inactivation photosensitized by 25 μM MC540 in distilled water ( *2* ) and in NaCl containing (0.25 M) solutions ( *1* ). Curves *3* and *4* correspond to the photoinactivation of *S. aureus * in distilled water and 0.25 M NaCl solutions without MC540, respectively.

 The absorption spectra of the MC540 water and water-salt solutions differ in both amplitude and shape. 

In the absorption spectra of a MC540 aqueous solution, there are two peaks ( *Fig. 1* , curve *1* ): at around 500 and 533 nm. These peaks are associated with dimers and monomers, respectively; they are referred to as “water peaks” [11]. When 0.25 M of sodium chloride is added to an aqueous solution of MC540, the water peaks disappear and a new band with a maximum at approximately 518 nm and two weak shoulders at around 570 and 620 nm occurs ( *Fig. 1* , curve *2* ). This new band appears as a result of the formation of aggregates [12], which is confirmed by the appearance of a band in the RLS spectrum [10] ( *Fig. 1* , curve *4* ). 

The RLS spectra measured under the same conditions as the absorption spectra are shown in *Fig. 1* ( *Fig. 1* , curves 3 and *4* ). In the absence of salt, a low-intensity band at around 550 nm is observed in the RLS spectrum ( *Fig. 1* , curve  *3* ); the appearance of this band is a result of the fluorescence of MC540 in the anti-Stokes region (0’–0-transition). In the absorption region of both the MC540 monomers and dimers, no resonance light scattering is observed. Resonance light scattering is observed for a 0.25 M NaCl solution of MC540, which confirms the formation of MC540 aggregates. The shape of the RLS spectrum resembles the shape of the measured absorption spectrum of a salt solution of MC540. The measured absorption spectrum of a water-salt solution of MC540 has an intense, symmetric and unstructured band with a maximum at around 518 nm and two less intense shoulders at 580 and 620 nm. The RLS spectrum is similar to the absorption spectrum. The most intense light scattering band overlaps with the most intense absorption band; however, the former band is slightly shifted towards the long-wavelength region and is found to have a fine structure with maximums at about 506 and 528 nm. On the long-wavelength side from the main RLS band, there is a tail observed in the absorption spectrum, as well ( *Fig. 1* , curve *4* ). 

The dependence of the RLS intensity on the concentration of NaCl added into a 7.6 µM solution of MC540 is shown in *Fig. 2* . It is clear that, at a salt concentration of less than 0.25 M, no RLS signal is observed. Upon further increase in the salt concentration, an abrupt growth in the intensity of light scattering occurs; this is indicative of the formation of dye aggregates. The concentration of the salt above which the formation of aggregates occurs was called the “critical aggregation concentration” (CAC). It should be noted that the CAC value does not change if we substitute NaCl for KCl; in other words, the critical aggregation concentration depends only on the valence of salt cations. The CAC values were determined for MC540 concentrations ranging from 5 to 25 µM. The dependence of the CAC value on the concentration of MC540 is presented in *Fig. 3* . According to these data, the CAC *vs.* MC540 concentration curve can be described as hyperbole and the [CAC] × [MC540] product remains constant within the entire concentration range studied; its value is (1.4 ± 0.05) × 10 ^–6^  M ^–2^ . This product is the solubility product of MC540 (like the solubility product of water). The data presented in *Fig. 3* allows us to calculate the fraction of the nonionized molecules of MC540, which form the extended aggregates. In 25 µM solutions of MC540 containing NaCl (0.25 M), this value exceeds 3/4 of the total amount of MC540 molecules. 

**Photobleaching of MC540**

**Fig. 7:**
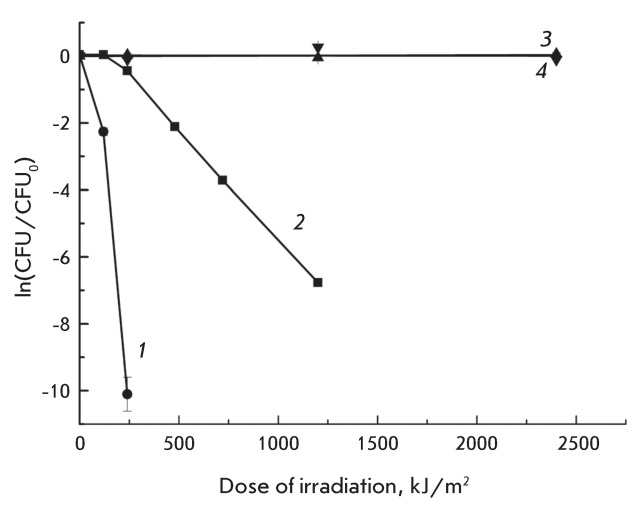
Dose-response curves of *P. aeruginosa* inactivation photosensitized by 25 μM MC540 in distilled water ( *2* ) and in NaCl containing (0.25 M) solutions ( *1* ). Curves *3* and *4* correspond to the photoinactivation of *P. aeruginosa * in distilled water and in  0.25 M NaCl solutions without MC540, respectively.

**Table 1:** Photoinactivation constants ( *β* , m ^2^ /kJ) for *P. aeruginosa * and *S. aureus* for 25 µM MC540 in distilled water and in NaCl-containing (0.25 M) solutions

*P. aeruginosa 104*		*S. aureus 78 *	
in water	in 0.25 M NaCl	in water	in 0.25 M NaCl
(6700±600) × 10^-6^	(66900±2500) × 10^-6^	(500±60) × 10^-6^	(13800±600) × 10^-6^

 The process of photobleaching proceeds differently in an aqueous MC540 solution from the way it proceeds in a solution containing NaCl (0.25 M). 

In the absorption spectra of an aqueous MC540 solution, it is obvious that an increase in the dose of irradiation leads to a comparable drop in the optical density ( *Fig. 4A* ) of both “water peaks” corresponding to MC540 monomers and dimers, respectively; the shape of the spectrum in this case does not change within the studied range of doses. 

In the presence of NaCl, the rate at which MC540 photobleaches is significantly higher than in an aqueous solution ( *Fig. 4B* ). In addition, the shape of the absorption spectra changes during irradiation. After approximately 10 min of irradiation, disappearance of the peak at 518 nm becomes noticeable and the intensity of “water peaks” grows (the insertion in *Fig. 4B* ). 

From *Fig. 5* it follows that the beginning segment of the bleaching *vs. * dose curves constructed for water and water–salt (NaCl, 0.25 M) solutions of MC540 has the shape of a straight line in semilogarithmic coordinates and can consequently be described by a mono-exponential function. 

The photobleaching constants ( *k* ) were calculated for 25 µM MC540 solutions from the slope of the corresponding lines (see “Experimental”), and their values were as follows: (70 ± 3) × 10 ^–6^  m ^2^ /kJ for the distilled water solution and (2080 ± 80) × 10 ^–6^  m ^2^ /kJ for the water solution containing sodium chloride (0.25 M). It is obvious that the rate of MC540 photobleaching is 30 times higher in the presence of NaCl in a water solution compared to a solution containing only distilled water. In a salt solution, MC540 is encountered mainly in its aggregated state, which in turn defines the optical density of the solutions. Accordingly, the reason for the higher rate of MC540 photobleaching observed in a salt solution in comparison with a distilled water solution could be the higher photolability of MC540 aggregates, rather than the dimers and monomers. 

The curves, in semilogarithmic coordinates, which characterize how the dose affects MC540 photosensitized inactivation of *S. aureus* in distilled water and NaCl containing (0.25 M) water solutions, are shown in *Fig. 6* ( *Fig. 6* , curve  *1* ). 

Irradiating cells in the absence of the photosensitizer caused inactivation neither in the distilled water solution ( *Fig. 6* , curve  *3* ) nor in the 0.25-M NaCl solution ( *Fig. 6* , curve *4* ). In the preliminary experiments, it was established that the incubation of *P. aeruginosa* and *S. aureus* cells in a 25 µM MC540 solution without irradiation had no effect on their survival (data not presented). 

The rate constants for MC540 photosensitized inactivation of bacteria are listed in *Table* ; the values were calculated as described in “Experimental.” According to these data, MC540 photosensitized inactivation of *S. aureus* proceeds faster in the presence of salt as compared to a distilled water solution. 

The dose dependences of MC540 photosensitized inactivation of *P. aeruginosa* in distilled water and in a 0.25 M NaCl solution are shown in Fig. 7 (curves  *1* and *2* , respectively). Irradiation in the absence of MC540 caused inactivation neither in distilled water (curve 3) nor in a 0.25 M NaCl solution (curve  *4* ). 

Under irradiation in the presence of MC540, in the initial segments of the doseresponse curves, a shoulder was observed when no inactivation of *P. aeruginosa* had occurred ( *Fig. 7* , curves  *1* and *2* ). The initial nonlinear segments of curves *1* and *2* ( *Fig. 7* ) are most likely related to the repair of photodamage; the processes compensate for the damage caused by antimicrobial photodynamic therapy at the early stages of photoinactivation. At high doses of irradiation, the inactivation curves displayed exponential behavior and assumed the shape of a straight line in semilogarithmic coordinates. The extrapolation of the linear segments before their intersection with the horizontal line corresponding to ln( *CFU* / *CFU*_0_ ) = 0 enabled us to determine the doses for which linear regions occur. These doses were called thresholds ( *F*_th_ ). For water suspensions, *F*_th.water_  = 140 kJ/m ^2^ , and for cell suspensions in a salt solution, *F*_th.salt_  = 81 kJ/m ^2^ . Thus, we introduced this variation, which takes into account the dose threshold value, into the equation for calculating the photoinactivation constant: 

*β* = ln( *CFU* / *CFU*_0_ )/( *F*  –  *F*_th_ ), 

where *CFU*_0 _ and *CFU* are the parameters that characterize the ability to form colonies at the initial time and under irradiation at a dose (kJ/m ^2^ ) of ( *F * –  *F*_th_ ) , respectively. 

The values of the photoinactivation constants are listed in *Table* . 

It can be observed that the photosensitized inactivation of *P. aeruginosa* proceeds 10 times more effectively in the presence of salt than it does in just distilled water. 

## DISCUSSION

It is known that the aggregation state of MC540 defines the type of photodynamic reactions that occur and affects the formation of active products, which can inflict damage on biological molecules [13] and can also influence the rate of MC540 photobleaching [7, 8]. The production of singlet oxygen ( ^1^ O _2_ ) was believed to play the main role in the bactericidal activity of photosensitizers [5, 14]. At the same time, it was shown that singlet oxygen can generate only monomeric forms of MC540 [15]. 

In this work, we studied how the aggregation state of MC540 affects its rate of photobleaching in the presence of 0.25 M NaCl and the rate of MC540 photosensitized inactivation of bacteria. The calculations that we performed relying on the data from *Figs. 2 * and * 3 * reveal that, at the given concentration of sodium chloride and MC540, 3/4 of the dye molecules are found in the aggregated state. In previously published works devoted to the study of the MC540 photosensitized inactivation of bacteria, no attempts were made to influence the aggregation state of this dye and by this the efficiency of inactivation [2, 3]. 

MC540 is an anionic photosensitizer. In distilled water, this compound exists in the form of dimers and monomers, which have absorption peaks at 533 and 500 nm, respectively [11]. Since the cell wall of bacteria is negatively charged [16–19], just as MC540 dimers and monomers, the penetration of the photosensitizer through the bacterial cell wall is hindered by electrostatic repulsion; in this case, the efficiency of MC540 photosensitized inactivation of bacteria decreases. However, when salt is added to the solution, its cations shield the anionic group of MC540; the latter leads to a reduction in the electrostatic repulsion between the molecules of MC540 and, thereby, to the formation of extended MC540 aggregates, which are detected by the RLS method. In addition, salt cations shield negative charges on the bacterial cell wall; this may facilitate the interaction of the photosensitizer with bacteria.

In our work, we demonstrated by means of the RLS method that when salt is added to an aqueous solution of MC540, there is a critical aggregation concentration (CAC) of salt above which aggregation of MC540 occurs; the CAC value depends on the concentration of MC540 ( *Fig. 2* ). The product of the CAC and the MC540 concentration in solutions of monovalent cations remains constant, and its value is [CAC] × [MC540] = (1.4 ± 0.05) × 10 ^–6^  M ^2^ . 

The results obtained in this work show that the rate of photobleaching of a 25 µM MC540 in a NaCl containing solution is 30 times higher than that in a distilled water solution; the rate constants of photobleaching are (2080 ± 80) × 10 ^–6^ and (70 ± 3) × 10 ^–6^ m ^2^ /kJ, respectively. In the case of MC540 photosensitized inactivation of *P. aeruginosa* , the photoinactivation constant increases by a factor of ten in the presence of sodium chloride (0.25 M), in comparison with its value in distilled water; the values of the photoinactivation constants are (66900 ± 2500) × 10 ^–6^ and (6700 ± 600) × 10 ^–6^ m ^2^ /kJ, respectively. In the case of *S. aureus* , the photoinactivation constant in the presence of 0.25 M NaCl is approximately 28 times higher [(13800 ± 600) × 10 ^–6^ m ^2^ /kJ] than the inactivation constant in distilled water [(500 ± 60) × 10 ^–6^ m ^2^ /kJ]. We suggest that such a difference in the rate constants of photobleaching ( *k* ) and photoinactivation ( *β* ) can be accounted for by the influence of salt cations on both the photosensitizer and the bacterial cell wall. 

Only monomers of MC540 possess the ability to generate ^1^ O _2_ [14]. During aggregation, the concentration of monomers drastically decreases; consequently, the increase in the bactericidal activity occurring in the presence of sodium chloride cannot be associated with the prevailing effect of ^1^ O _2 _ on bacteria. It may be suggested that the transfer of an electron between the excited and unexcited molecules of the dye is facilitated in the aggregates of MC540. In all likelihood, the presence of the salt causes the activation of photodynamic reactions in the aggregates of MC540, which results in the generation of free radicals [13], the latter having the ability to attack bacteria and, subsequently, to kill them. 

## CONCLUSIONS

The data obtained in this work indicating an increase in the bactericidal effects of a photosensitizer in the presence of salts can be used in the development of promising new antibacterial treatments especially in light of the current problems connected with multiple antibiotic resistance. 

## Abbreviations

CAC: critical aggregation concentration
MC540: merocyanine 540
RLS: resonance light scattering
PS: photosensitizer
CFU: colony-forming unit
